# CMV Latent Infection Improves CD8+ T Response to SEB Due to Expansion of Polyfunctional CD57+ Cells in Young Individuals

**DOI:** 10.1371/journal.pone.0088538

**Published:** 2014-02-12

**Authors:** Alejandra Pera, Carmen Campos, Alonso Corona, Beatriz Sanchez-Correa, Raquel Tarazona, Anis Larbi, Rafael Solana

**Affiliations:** 1 Department of Immunology, Maimonides Institute for Biomedical Research (IMIBIC) – Reina Sofía University Hospital – University of Cordoba, Cordoba, Spain; 2 Immunology Unit, Department of Physiology, University of Extremadura, Cáceres, Spain; 3 Singapore Immunology Network (SIgN), Biopolis, Agency for Science, Technology and Research, Singapore, Singapore; Karolinska Institutet, Sweden

## Abstract

Cytomegalovirus (CMV) latent infection has a deleterious effect on the efficacy of influenza vaccination in the elderly, suggesting that CMV restricts immunological diversity impairing the immune system functionality in old age. Polyfunctional T cells produce multiple cytokines and higher amounts than mono-functional T cells. High number of polyfunctional T cells correlates with better prognosis during infection. Thus, the efficiency of T cell response associates with quality (polyfunctionality) rather than with quantity (percentage of T cells). We analyze the effect of CMV infection on CD8+ T cells polyfunctionality ―degranulation (CD107a), IFN-gamma and TNF-alpha production―, from young CMV-seropositive and CMV-seronegative individuals and in middle age CMV-seropositive donors, in response to Staphylococcal Enterotoxin B (SEB). Our results show a higher percentage of polyfunctional CD8+ T cells in young CMV-seropositive individuals compared to CMV-seronegative. Also, we find an expansion of CD8+CD57+ T cells in CMV-seropositive individuals, which are more polyfunctional than CD8+CD57− cells. In middle age individuals there is a higher frequency of SEB-responding CD8+ T cells, mainly TNF-alpha or TNF-alpha/IFN-gamma producers, whereas the percentage of polyfunctional cells (IFN-gamma/TNF-alpha/CD107a) is similar to the percentages found in young CMV-seropositive. Therefore, whereas it has been shown that CMV latent infection can be detrimental for immune response in old individuals, our results indicate that CMV-seropositivity is associated to higher levels of polyfunctional CD8+ T cells in young and middle age donors. This increase in polyfunctionality, which can provide an immunological advantage in the response to other pathogens, is due to a CD8+CD57+ T cell expansion in CMV-seropositive individuals and it is independent of age. Conversely, age could contribute to the inflammation found in old individuals by increasing the percentage of cells producing pro-inflammatory cytokines. These findings highlight the necessity of further studies on the benefits/detrimental effects of CMV infection in the response to vaccination and other infections.

## Introduction

CMV chronic infection has a high prevalence that varies worldwide. Seropositivity is related to geographic, ethnic and social factors and increases with age [1]. In Spain up to 80% of individuals over the age of 40 years are CMV-seropositive [2]. Primary CMV infection takes place generally during puberty, after which the virus endures, controlled by constant surveillance of the immune system [3], [4]. In most cases, CMV infection is subclinical and well tolerated, even though latent infection is associated with an age-related deterioration of the immune system, in particular CD8+ T cell compartment, causing a distortion of T cell repertoire with expansions of CMV-specific CD8+ T cells that can represent up to 20% of total CD8+ T cell population [5]–[7]. CMV-seropositivity is also associated with an increased risk of death and cardiovascular diseases [8]–[10] and with the development of an “Immune Risk Phenotype” (IRP), predictive of early mortality in the elderly [11]–[13]. Thus, CMV is considered a major driving force of immunosenescence characterized by the accumulation of antigen-specific, oligoclonally expanded CD8+CD28–CD57+ T cells. These cells have been proposed as terminally differentiated, senescent, dysfunctional, effector-memory T lymphocytes that have gone through several rounds of cell divisions (for review see [14], [15]).

It has been suggested that the negative impact of CMV seropositivity over survival in the elderly could be due, at least in part, to the limitation in the T cell repertoire caused by the expansion of dysfunctional and highly differentiated or senescent CMV specific T cells, that affects the functional abilities of other T cell populations combating new infections and/or tumors [16].

It has been also shown that latent mouse CMV (MCMV) infection impairs immunity to other viruses in old age and increases the percentages and absolute numbers of CD8+ and effector-memory CD8+ T cells, thus contributing to immunosenescence [17], [18]. However, evidences from other studies in young mice have shown that latent MCMV infection contributes to immune protection against infection with unrelated pathogens [19], [20]. These apparently contradictory results make necessary to analyze how CMV infection can affect the immune response in young humans.

It is well known that a single T cell can produce simultaneously multiple cytokines, being then commonly referred as polyfunctional. Several publications have shown that a higher number of polyfunctional T cells is correlated with a better prognosis during HIV infection [21] and better response to vaccination in animals [22], indicating that the efficiency of the response is associated with the capacity of responding cells to produce several cytokines (“polyfunctionality” as a marker of quality) rather than with the percentage (quantity) of specific T cells.

It has been shown that CMV-specific CD8+ T cells from elderly donors retain their proliferative and cytotoxic capacity [23] and that T cells responding to CMV are polyfunctional independently of donor age [24]. Moreover, a recent study does not find differences in the response to West Nile virus in a group of middle age and aged individuals with large expansions of CMV-reactive CD8+ T cells, indicating that CMV infection and the subsequent expansion of CMV-specific CD8+ T cells do not limit the ability of the host to respond to novel antigens [25].

However, whether CMV infection in young individuals affects the polyfunctionality of CD8+ T lymphocytes in response to other antigens has not yet been studied. The aim of this work is to analyze the effect of CMV seropositivity and age on polyfunctionality of CD8+ T cells in response to the super-antigen Staphyloccocal Enterotoxin B (SEB). To this end, we analyzed CD8+ T cells polyfunctionality (degranulation, IFN-gamma and TNF-alpha production) from young healthy individuals stratified by CMV status and from middle age CMV-seropositive individuals. The results obtained demonstrate that there is a higher frequency of polyfunctional CD8+ T cells in response to SEB stimulation in young and middle age CMV-seropositive donors, compared with CMV-seronegative donors, which is associated to the expansion of polyfunctional CD8+CD57+ T cells.

## Results

### Effect of age and CMV infection on CD8+ T cell response to SEB stimulation

**Figure 1:**
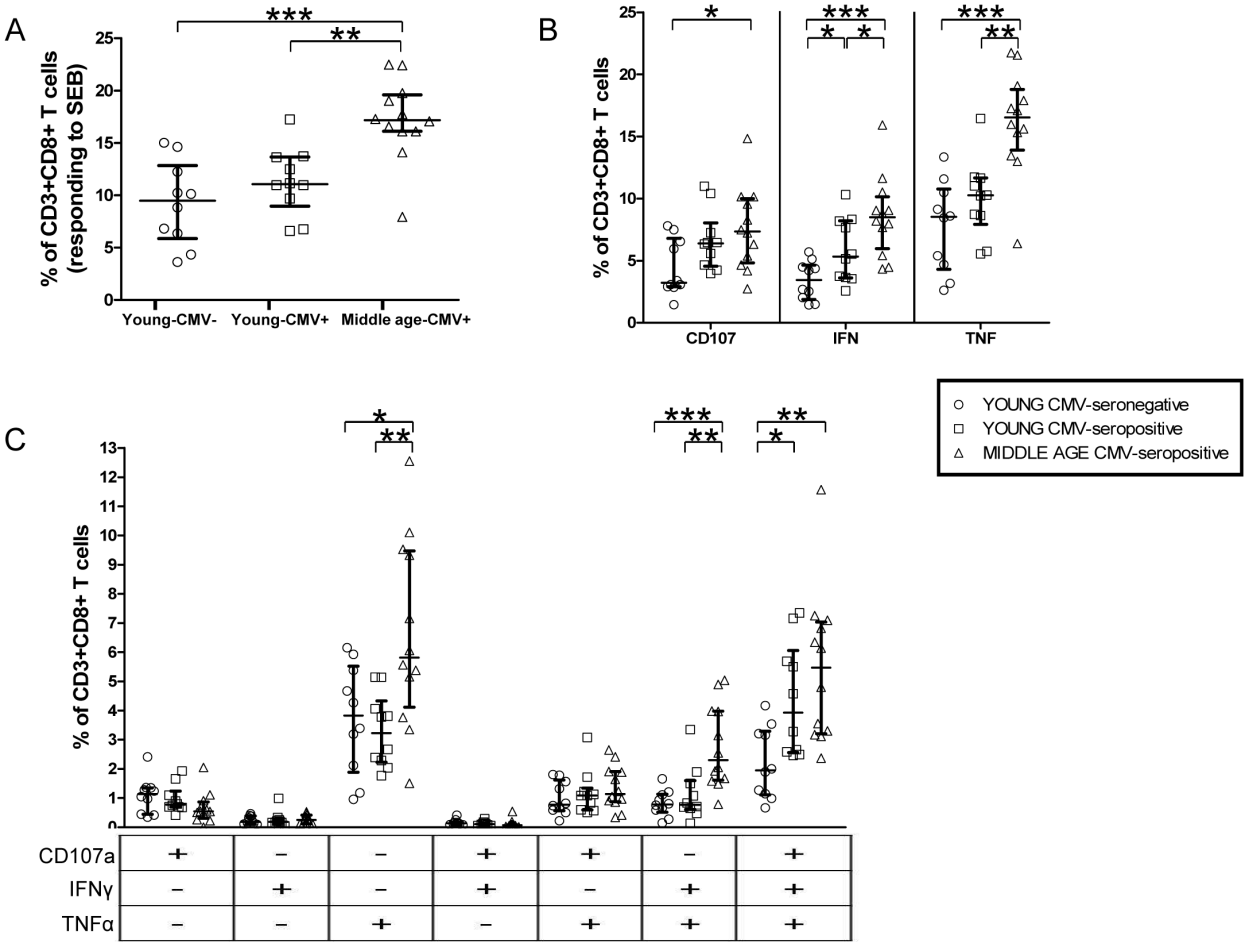
CD8+ T cells' responses to SEB stimulation, in healthy individuals stratified by CMV serostatus and age. A) Percentage of CD3+CD8+ T cells that have any studied response to SEB. B) Total degranulation (CD107a) and cytokine production (IFN-gamma, TNF-alpha) by CD3+CD8+ T lymphocytes responding to SEB. Vertical black lines indicate interquartile ranges, ranging from the 25th to the 75th percentile. The median response for each category is indicated by a horizontal black line. C) Three-function analysis of SEB responses. Scatter graphs show the magnitude of SEB responses in each functional category, expressed as percentage of CD3+CD8+ T cells. The combination of functions studied is indicated in the table below the scatter graphs.

We analyzed IFN-gamma and TNF-alpha production as well as the expression of CD107a, —a component of cytolytic granules, which is used as a marker of CD8+ T cell degranulation after activation— in CD8+ T cells from young CMV-seronegative individuals, young CMV-seropositive and middle age individuals. The percentage of CD8+ T cells was similar in the three groups studied. The analysis of the frequency of SEB-responding CD8+ T cells, considered as those capable of any kind of response (CD107a, IFN-gamma, TNF-alpha), showed that the percentage of SEB-responding CD8+ T cells is higher in middle age CMV-seropositive individuals compared to young individuals, both CMV-seropositive and CMV-seronegative. On the contrary, no significant differences were observed between young CMV-seronegative and CMV-seropositive individuals (Figure 1A).

When we analyzed each kind of response individually, we observed that the percentage of IFN-gamma producing CD8+ T cells was higher in young CMV-seropositive individuals compared to young CMV-seronegative (Figure 1B). Middle age CMV-seropositive individuals had a higher percentage of cytokine producing CD8+ T cells (TNF-alpha or IFN-gamma) compared to young donors (either CMV-seronegative or CMV-seropositive). Besides, CD107a expression was higher in middle age CMV-seropositive individuals when compared to young CMV-seronegative individuals.

Analysis of CD8+ T cells polyfunctionality was performed as described in materials and methods (Figure S1). The percentage of CD8+ T cells producing only IFN-gamma or IFN-gamma in combination with CD107a (IFN-gamma/CD107a) in response to SEB was noticeably low or null in all subjects studied, independently of age or CMV-serostatus. In fact, CD8+ T cells producing IFN-gamma were basically bi- or tri-functional (IFN-gamma/TNF-alpha; CD107/IFN-gamma/TNF-alpha) in the three groups studied (Figure 1C). Of notice is the increase of tri-functional CD8+ T cells found in CMV-seropositive (young and middle age) when compared to young CMV-seronegative individuals. However, no significant differences were found in the percentage of tri-functional CD8+ T cells between young and middle age CMV-seropositive individuals, indicating that this increase is associated mainly to CMV infection (Figure 1C). TNF-alpha production was the single strongest response in the three groups studied. The percentage of TNF-alpha producers within CD8+ T cells (TNF-alpha and IFN-gamma/TNF-alpha producing cells) was significantly higher in middle age CMV-seropositive individuals (Figure 1C).

Thus, CMV-seropositivity in young individuals is associated to higher levels of polyfunctional CD8+ T cells that are maintained in middle age. In addition, middle age donors have a higher percentage of SEB-responding CD8+ T cells that are mainly TNF-alpha single-producers or TNF-alpha and IFN-gamma double-producers.

### CD8+CD57+ T cells are more polyfunctional than CD8+CD57– and are increased in CMV-seropositive individuals

**Figure 2:**
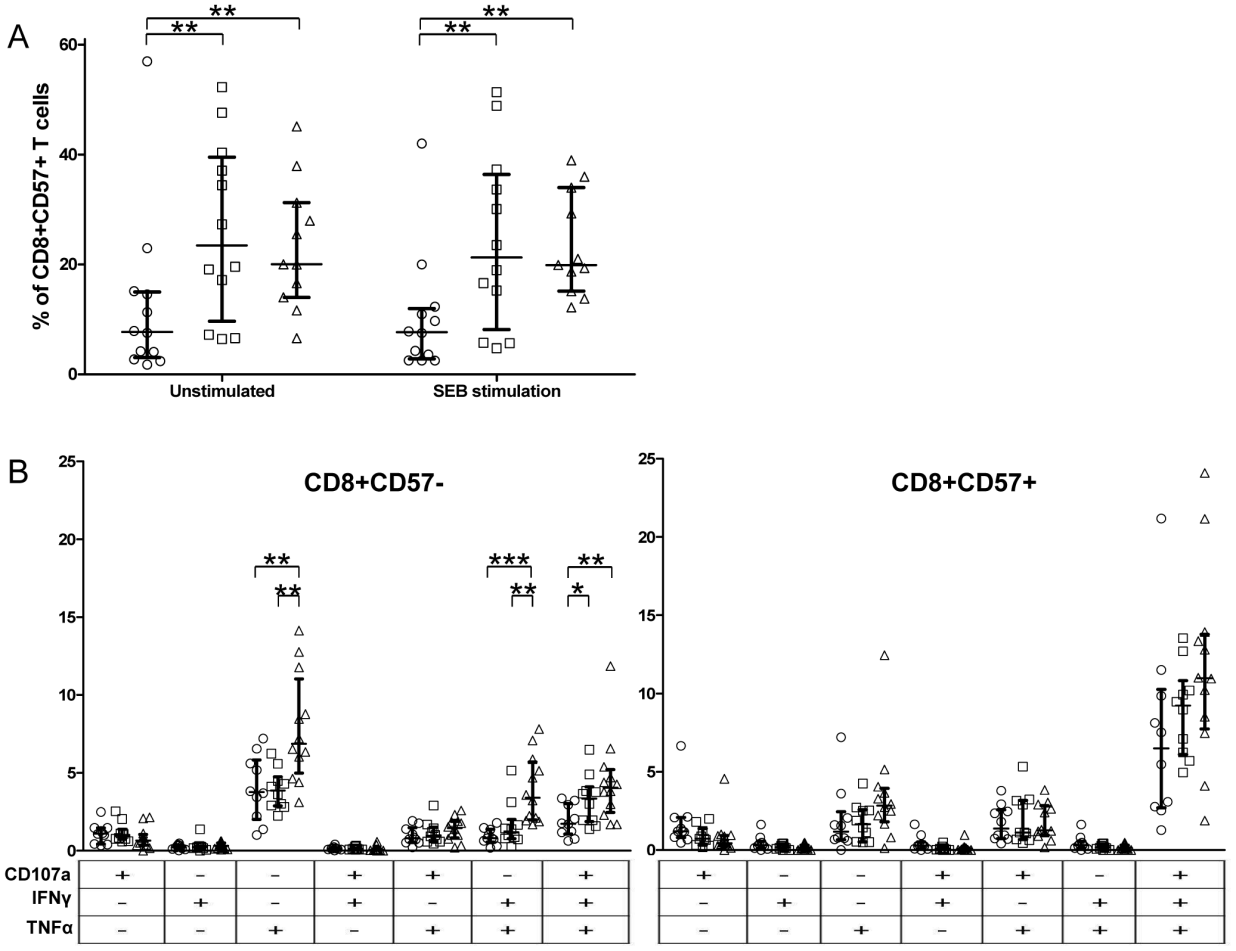
CD8+CD57+ T cell frequency and functionality in healthy individuals. A) CD57 expression in CD8+ T cells of healthy individuals stratified by CMV serostatus and age. B) Three-function analysis of SEB responses of CD8+CD57– and CD8+CD57+ T cell subsets. Scatter graphs show the magnitude of SEB responses in each functional category, expressed as percentage of CD8+CD57- T cells or CD8+CD57+ T cells. Vertical black lines indicate interquartile ranges, ranging from the 25th to the 75th percentile. The median response for each category is indicated by a horizontal black line. The combination of functions studied is indicated in the table below the scatter graphs.

CD8+CD57+ T cell percentages were determined by gating on resting CD3+ cells. The results showed a significant increase of CD8+CD57+ T cell subset in young and middle age CMV-seropositive individuals compared to young CMV-seronegative. No significant differences on CD57 expression were observed between resting and SEB stimulated CD8+ T cells (Figure 2A).

The analysis of the polyfunctional capacity of CD8+ T cells responding to SEB in relation to CD57 expression in all individuals showed a significant increase of tri-functional CD8+CD57+ T cells and a lower frequency of single TNF-alpha and double IFN-gamma/TNF-alpha producer CD8+CD57+ T cells compared with CD8+CD57– T cells (Figure S2). These differences between CD8+CD57+ and CD8+CD57– T cells were also observed when donors were stratified according to CMV serostatus (CMV-seronegative, CMV-seropositive) and/or age (young, middle age) (Figure S3). In fact, CD8+CD57+ responding T cells were mostly polyfunctional (CD107a/IFN-gamma/TNF-alpha), while CD8+CD57– responding T cells were mainly mono-functional (Figure 2B).

**Figure 3:**
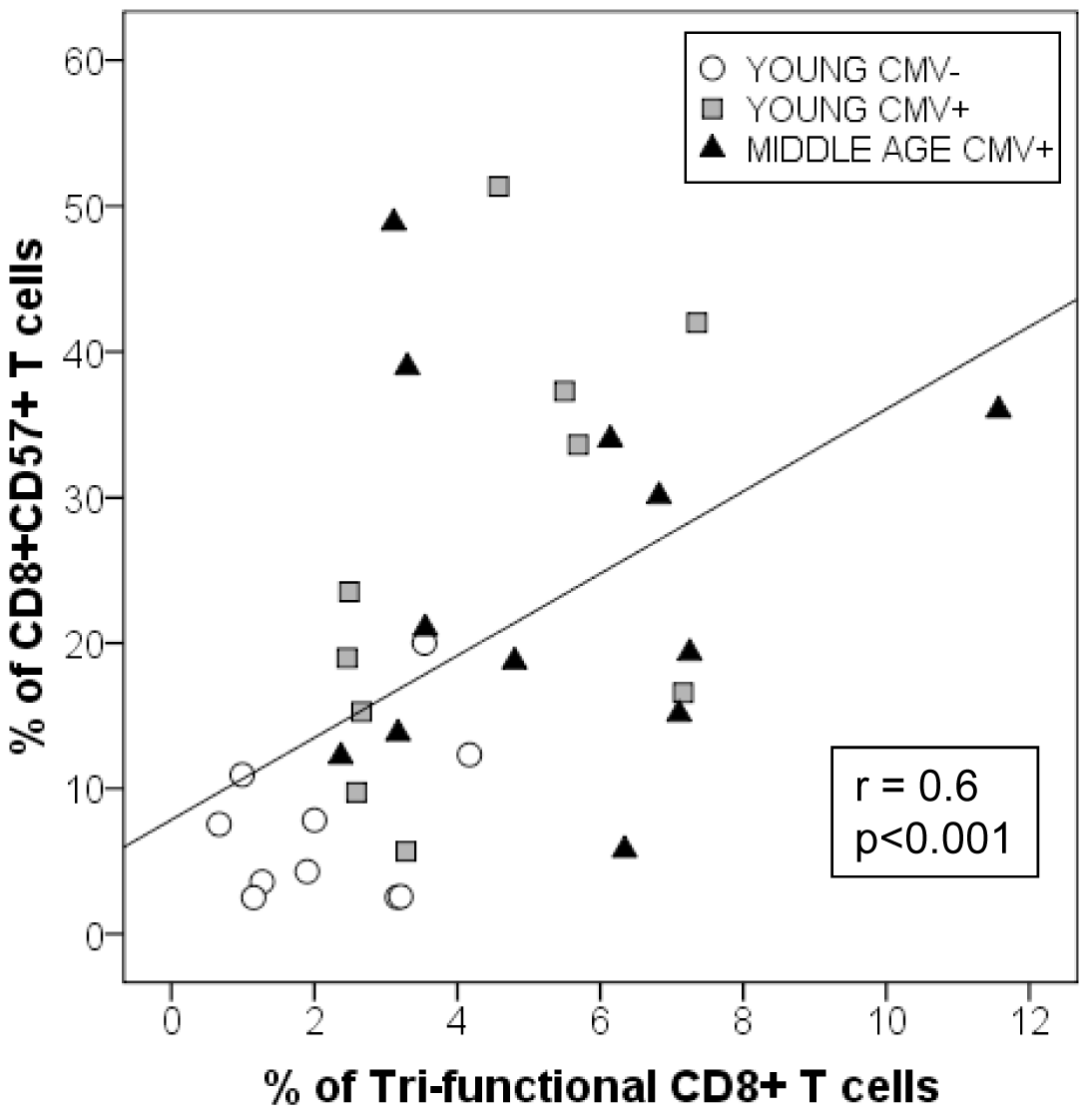
Correlation between tri-functional CD8+ T cells and CD57 expression in the individuals studied (n = 32). Y axis represents the percentage of CD8+ T cells that express CD57. X axis represents the percentage of tri-functional CD8+ T cells.

Analysis of CD8+CD57+ T cell functionality did not show significant differences either with age or CMV seropositivity (Figure 2B). Moreover, there was a significant correlation between the percentage of CD8+CD57+ T cells and the percentage of tri-functional CD8+ T cells (Figure 3), indicating that CMV seropositivity increased CD8+ T cell polyfunctionality in young individuals was due to the expansion of polyfunctional CD8+CD57+ T cells in these individuals.

When the functional capacity of CD8+CD57– T cells was compared among the three groups studied, we observed a higher percentage of TNF-alpha and IFN-gamma/TNF-alpha producing cells in middle age CMV-seropositive individuals compared to young donors (CMV-seropositive and CMV-seronegative). Thus, the higher response of CD8+ T cells found in middle age CMV-seropositive individuals when compared with young CMV-seropositive individuals (Figure 1A, B) was due to a higher percentage of non-cytotoxic (CD107a–) CD8+CD57- T cells that responded to SEB by producing cytokines (TNF-alpha alone or IFN-gamma/TNF-alpha) (Figures 2B, S2 and S3). It is of notice that bi-functional IFN-gamma/TNF-alpha CD8+ T cells were only found in the CD57- subset (Figure S2). With respect of CMV seropositivity, there was a higher frequency of tri-functional CD8+CD57– T cells in CMV-seropositive individuals (young and middle age) compared with young CMV-seronegative (Figure 2B).

In summary, CMV seropositive donors have higher numbers of polyfunctional CD8+ T cells associated to the expansion of polyfunctional CD8+CD57+ T cells. Middle age individuals have similar percentages of polyfunctional CD8+ T cells (both CD57- and CD57+) and CD8+CD57+ T cells than young CMV-seropositive donors. However, middle age CMV-seropositive individuals have a higher frequency of CD8+CD57- T cells that produce TNF-alpha alone or in combination with IFN-gamma.

## Discussion

To the best of our knowledge, this is the first description that young CMV-seropositive individuals have a higher percentage of tri-functional CD8+ T cells in response to SEB, compared with young CMV-seronegative individuals. This increase is associated with the expansion of CD8+CD57+ T cells observed in CMV-seropositive individuals that are mainly tri-functional and to some extent to the higher levels of tri-functionality in the CD8+CD57− T cell subset. Furthermore the percentages of tri-functional CD8+ T cells and CD8+CD57+ T cells are similar among young and middle age CMV−seropositive individuals. On the contrary, the frequencies of CD8+CD57− T cells producing TNF-alpha or IFN-gamma/TNF-alpha are significantly higher in middle age compared with young CMV-seropositive donors.

It has been demonstrated that protection against pathogens is related to T cell quality (polyfunctionality) rather than to the frequency of reactive T cells or the amount of the T cell response [26], [27]. Moreover, polyfunctional T cells produce higher amounts of cytokines than mono-functional T cells, i.e., polyfunctional cells are more efficient [24]. Thus our observation that young CMV−seropositive subjects have an increased percentage of tri-functional CD8+ T cells in response to SEB indicates that CMV seropositivity improves the quality of the response to SEB. These data support the possibility that CMV latency promotes the response to non-related antigens through heterologous immunity. Heterologous immunity refers to the protection that a host can develop against one pathogen after being exposed to other non-identical pathogens [28]. In particular, murine herpesviruses provide heterologous immunity against other viruses and bacteria and it has been suggested that herpesvirus latency has coevolved to promote host immunity to infectious diseases [20]. Although most studies on heterologous immunity have been performed in murine models, there are evidences that it can also occur in human [28]. In particular it has been shown that CMV infection enhances the immune response against measles vaccination in Gambian infants [29].

Our results show a CD8+CD57+ T cell expansion in CMV-seropositive individuals (young and middle age) compared to young CMV-seronegative. The expansion of CD8+CD57+ T cells has been described in different conditions associated to chronic immune stimulation such as virus infections, transplantation, autoimmune diseases and ageing (for review see [25], [30], [31]). The expansion of CD57+ cells in these clinical conditions has been associated with lower expression of CD28 and replicative senescence with a decreased proliferative capacity [32], [33]. However, there is evidence that CD8+CD57+ T cells proliferate under certain conditions [34]. In addition, it has been shown that these cells are mainly cytotoxic and produce IFN-gamma [35], [36]. In our hands, CD8+CD57+ T cells are mostly tri-functional and the higher frequency of SEB-responding polyfunctional CD8+ T cells found in CMV-seropositive individuals is mainly due to the expansion of these CD8+CD57+ T cells.

CD8+CD57+ T cells (or CD8+CD28− T cells) expand with ageing [30], [31]. Considering that the frequency of CMV-seropositive individuals increases with age in different social and ethnic groups [1], our results support the possibility that the major factor involved in this expansion is CMV latent infection rather than ageing. In addition it has been reported the expansion of these cells in several clinical situations characterized by chronic activation of the immune system [30], [37]. However, CMV seropositivity has not been analyzed in the majority of these studies, so the possible involvement of CMV in the increased percentage of CD8+CD57+ T cells cannot be excluded.

IFN-gamma production by CD8+CD57+ T cells has been suggested to play a detrimental role contributing to age-associated inflammatory diseases [38]. However, since our data show that IFN-gamma production by CD8+CD57+ T cells is a marker of CD8+ T cell polyfunctionality, it should be considered that CD8+CD57+ T cells play a beneficial role in the immune response against pathogens. On the contrary we have observed a higher percentage of CD8+CD57− T cells producing TNF-alpha alone or TNF-alpha and IFN-gamma in middle age individuals. Considering the relevance of TNF-alpha not only in inflammation but also in superantigen-induced toxic shock, we suggest that this cytokine pattern in CMV-seropositive individuals might be involved in age-associated inflammatory diseases. In addition, the higher levels of cytokines, in particular TNF-alpha, produced in response to SEB in CMV-seropositive individuals might contribute to toxic shock induced by bacterial superantigens.

It has been proposed that CMV is a major driving force of human T cell immunosenescence [5], [37], [39], [40]. It is well established that CMV infection has a deleterious effect on the efficacy of influenza vaccination both in young and elderly individuals [41], suggesting that CMV chronic infection restricts immunological diversity and impairs the immune system functionality. However, a recent study does not find differences in the response to West Nile virus in a group of middle age and aged individuals with large expansions of CMV-reactive CD8+ T cells, indicating that CMV infection does not limit the ability of the host to respond to novel antigens [25].

In summary our results showing that CMV infection is associated to a higher frequency of polyfunctional CD8+CD57+ T cells support the hypothesis that herpesvirus latency contributes to protection against some pathogens [19], [20]. As pointed out by Unanue [42] throughout evolution, latent herpesvirus infection could provide an immunological advantage, by keeping the immune system in a state of alert, which could explain the high prevalence of these viruses, in particular CMV, in humans. Thus in developing countries the cross-protection provided by CMV could be important to overcome other viral and bacterial infections in early years. However, surviving to infections in the infancy is not now a major issue in developed countries, whereas a lifelong activated immune system can trigger aberrant immune inflammatory responses that could lead to age-associated diseases. Therefore, the balance between the possible positive and negative effects of CMV should be considered in CMV vaccine studies. These facts raise the issue of the possible benefit or detriment from CMV eradication. Our results highlight the requirement of further studies to analyze how CMV latent infection affects the immune response to vaccination and to other infections in all groups of age, not only in the elderly but also in the infancy and in the young, in different socio-economic population groups.

## Materials and Methods

### Ethics statement

The study was approved by the Ethics Committee of the Reina Sofia University Hospital. All study participants provided informed written consent.

### Subjects

We studied 32 healthy donors; 20 young individuals (18–35 years old) stratified by CMV serostatus –10 CMV-seronegative (4 male/6 female; mean age 26.3, SD = 5.8) and 10 CMV-seropositive (6 male/4 female; mean age 27, SD = 4.3) – and 12 middle age individuals all of them CMV-seropositive (3 male/9 female; 40–60 years old, mean age 50.25, SD = 4.3). Due to the high prevalence of CMV in Spain we could not recruit enough middle age CMV-seronegative individuals (over 80% in older than 40 years old [2]). All subjects studied met the following exclusion criteria: absence of diabetes, cancer, severe renal failure, severe liver disease, endocrine disorders, autoimmune diseases, or acute infectious disease; they were not consuming drugs whose activity is known to modify the functions of the immune system.

Peripheral blood from each subject was collected by venipuncture in Lithium Heparin-containing tubes. Within 1–3 h after collection PBMCs were isolated from each blood sample using Ficoll-Histopaque density gradient centrifugation (Sigma Aldrich. Steinheim, Germany) and cryopreserved until the time the experiments were performed.

### CMV serology

Plasma or sera from all donors were tested for CMV-specific IgG and IgM by automated CMV enzyme-linked immunosorbent assay (ELISA), (Genesis Diagnostics, Cambridge, UK).

### Stimulation, intracellular staining, and detection of CD107a expression

As CD107a expression has been directly correlated with CD8+ T cell cytotoxicity [43], [44], we used this marker for the degranulation assays.

Freshly thawed, cryopreserved PBMCs were resuspended at 1×10^6^ cells/ml in complete RPMI media (RPMI 1640 supplemented with 10% heat inactivated FCS, 100 U/ml penicillin G, 100 µg/ml streptomycin sulphate and 17 mM sodium glutamate) and rested overnight at 37°C in a standard incubator (humidified CO_2_ atmosphere).

The following day, cells were placed in a 96 well plate at 2×10^6^ cells/ml concentration (250 µl final volume). Costimulatory antibodies (anti-CD28 and anti-CD49d; 1 µg/ml each; BD Biosciences) and anti-CD107a-APC (BD Biosciences) were added to all wells. Staphylococcal Enterotoxin B superantigen (SEB, Sigma-Aldrich) was added at a final concentration of 1 µg/ml. For each individual a negative control, containing only anti-CD28 and anti-CD49d, was included to measure antigen-independent stimulation.

The plate was placed in a standard incubator (37°C, humidified CO^2^ atmosphere) and, after 1 h, each well received the addition of monensin (Golgistop, 0.67 µl/ml; BD Biosciences) and brefeldin A (Golgi Plug 1 µg/ml; BD Biosciences). Cells were then incubated for an additional 4 h. Following incubation, cells were washed twice with PBS (4°C) and stained with surface antibody (CD57-VioBlue, Miltenyi Biotec). Cells were then fixed and permeabilized with Cytofix/Cytoperm solution according to the manufacturer's instructions (BD PharMingen) and subsequently stained intracellularly with CD3-PerCP, CD8-APC-Cy7 (BD Biosciences), IFN-gamma-FITC and TNF-alpha-PE (Miltenyi Biotec) antibodies, since it is preferable to stain cells with anti-CD3 and anti-CD8 antibodies after they are permeabilized, to partially, if not fully, eliminate the reduced staining of these molecules on the cell surface of activated T cells due to surface down-modulation of these markers after activation [43]. For isotype controls we followed the same protocol as samples. All antibodies used in the experiment were titrated before use. Stained cells were analyzed by flow cytometry the following day.

### Flow cytometry and data analysis

Flow cytometric analysis was performed on a two-laser MACsQuant instrument (Miltenyi Biotech) using MACSQuantify software. For each sample, data from almost 40.000 events of CD3+CD8+ gate were collected. Files were gated on small lymphocytes (using forward vs side scatter). When indicated the expression of CD57 was analyzed on resting CD8+ T cells.

Degranulating and cytokine-producing populations were defined as the percentage of the CD107a, IFN-gamma or TNF-alpha events gated on CD3+CD8+ T cells. The gating strategy used for polyfunctionality (CD107a, IFN-gamma, TNF-alpha) measurements is illustrated in Figure S1.

To study polyfunctionality of CD57 expressing CD8+ T cells after SEB stimulation, CD3+CD8+ T lymphocytes were gated and then divided into four quadrants: CD57-CD107−, CD57−CD107+, CD57+CD107−, CD57+CD107+. Cytokine production of IFN-gamma and/or TNF-alpha was defined by gating on each of these four CD8+ T cells subsets. The settings for CD107a, IFN-gamma and TNF-alpha staining were defined on the basis of an isotype-matched negative control of irrelevant specificity in separate unstimulated culture.

### Statistical analysis

Net responses were calculated by subtracting values for the negative control from SEB responses. For direct comparison of three independent samples, Kruskal-Wallis H test (nonparametric test) was used. Mann-Whitney U nonparametric test was used to derive *p* values for comparing data among the specific sample pairs. For comparison between CD8+ T cells responses to SEB and CD57 expression in CD8+ T cells Spearman correlation test was used. All statistical tests were performed with PASW Statistics v18; *p* values ≤0.05 were considered significant.

## Supporting Information

Figure S1 **Flow cytometry gating strategy used in the analysis of polyfunctionality flow cytometry data.** Figure shows PBMCs of a CMV-seropositive young healthy individual, stimulated with SEB. After initial gating on lymphocytes, cells were then selected based on CD3+CD8+ staining and then divided into two gates CD107- and CD107+. Each gate was then analyzed into a four quadrant plot representing IFN-gamma and/or TNF-alpha responses (IFNg-TNFa-, IFNg+TNFa-, IFNg-TNFa+, IFNg+TNFa+). Values are referred to the total of CD8+ T cells.
(TIF)

Figure S2 **CD8+ T cell polyfunctionality in relation to CD57 expression.** Polyfunctionality flow cytometry analysis of CD8+CD57– and CD8+CD57+ T cell subsets, in response to SEB stimulation. This figure summarizes data for all 32 healthy individuals enrolled in this study. Scatter graphs show the magnitude of SEB responses in each functional category, expressed as percentage of CD8+CD57− T cells or CD8+CD57+ T cells. Vertical black lines indicate interquartile ranges, ranging from the 25th to the 75th percentile. The median response for each category is indicated by a horizontal black line. The combination of functions studied is indicated in the table below the scatter graphs. Panel A, cells responding to SEB stimulation. Panel B cells that do not respond to SEB stimulation.
(TIF)

Figure S3 **CD8+ T cell polyfunctionality, in relation to CD57 expression, in the different groups.** Each graph shows the polyfunctional responses to SEB of CD8+CD57– and CD8+CD57+ T cell subsets for each group studied (young CMV-seronegative, young CMV-seropositive and middle age CMV-seropositive). Scatter graphs show the magnitude of SEB responses in each functional category, expressed as percentage of CD8+CD57− T cells or CD8+CD57+ T cells. Vertical black lines indicate interquartile ranges, ranging from the 25th to the 75th percentile. The median response for each category is indicated by a horizontal black line. The combination of functions studied is indicated in the table below the scatter graphs.
(TIF)
