# Correction: CMV latent infection improves CD8+ T response to SEB due to expansion of polyfunctional CD57+ cells in young individuals

**DOI:** 10.1371/journal.pone.0096971

**Published:** 2014-04-29

**Authors:** The PLOS ONE Staff

The third author’s name is spelled incorrectly. The first and last names of this author have been swapped. The correct name is: Corona Alonso.
