# Supplementary figures and images for: CMV Latent Infection Improves CD8+ T Response to SEB Due to Expansion of Polyfunctional CD57+ Cells in Young Individuals
Source: PLoS One. 2014 Feb 12;9(2):e88538. doi: 10.1371/journal.pone.0088538 (PMC3922920; doi:10.1371/journal.pone.0088538)

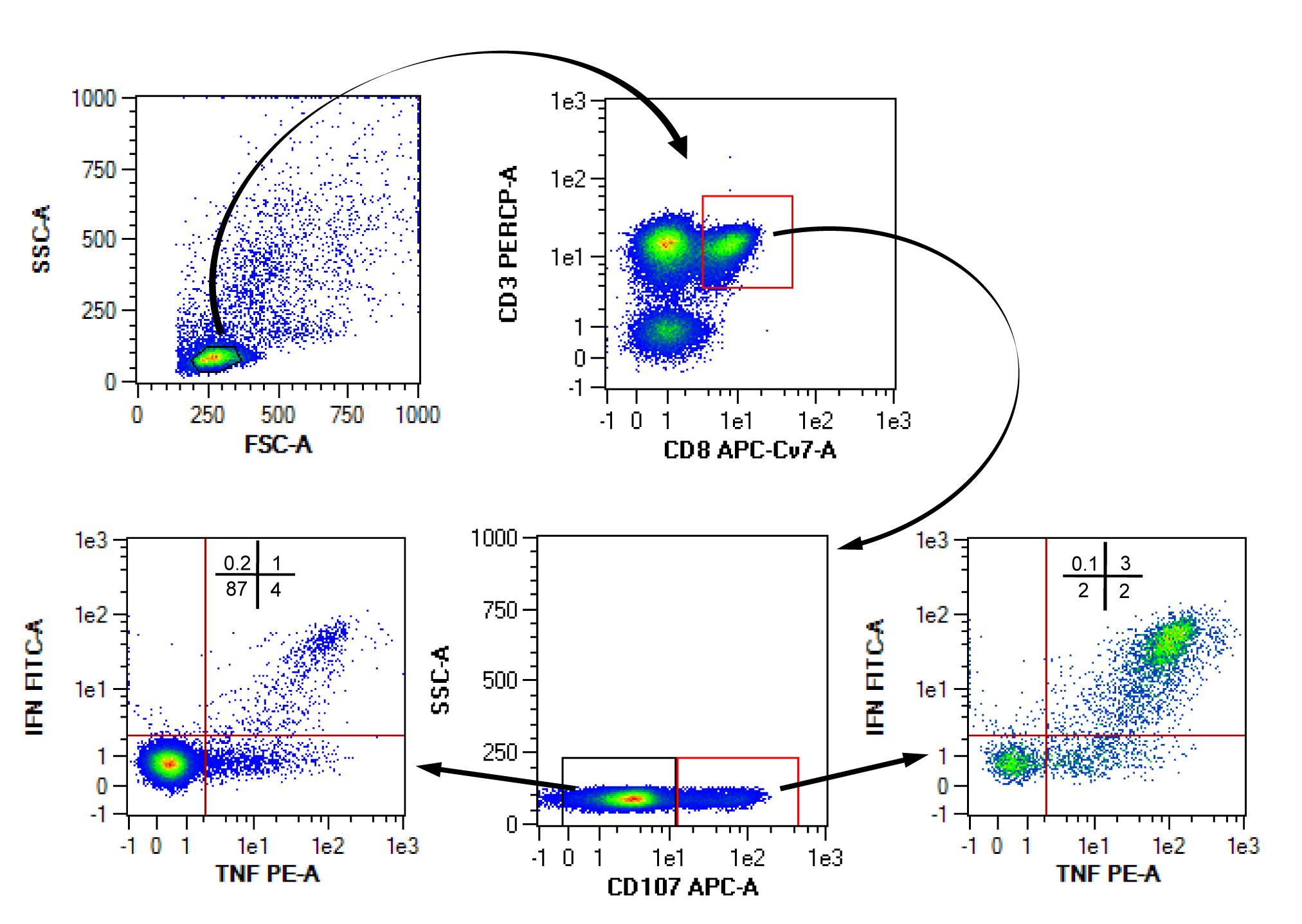

Supplement: Figure S1 — Flow cytometry gating strategy used in the analysis of polyfunctionality flow cytometry data. Figure shows PBMCs of a CMV-seropositive young healthy individual, stimulated with SEB. After initial gating on lymphocytes, cells were then selected based on CD3+CD8+ staining and then divided into two gates CD107- and CD107+. Each gate was then analyzed into a four quadrant plot representing IFN-gamma and/or TNF-alpha responses (IFNg-TNFa-, IFNg+TNFa-, IFNg-TNFa+, IFNg+TNFa+). Values are referred to the total of CD8+ T cells. (TIF) [file pone.0088538.s001.tif]

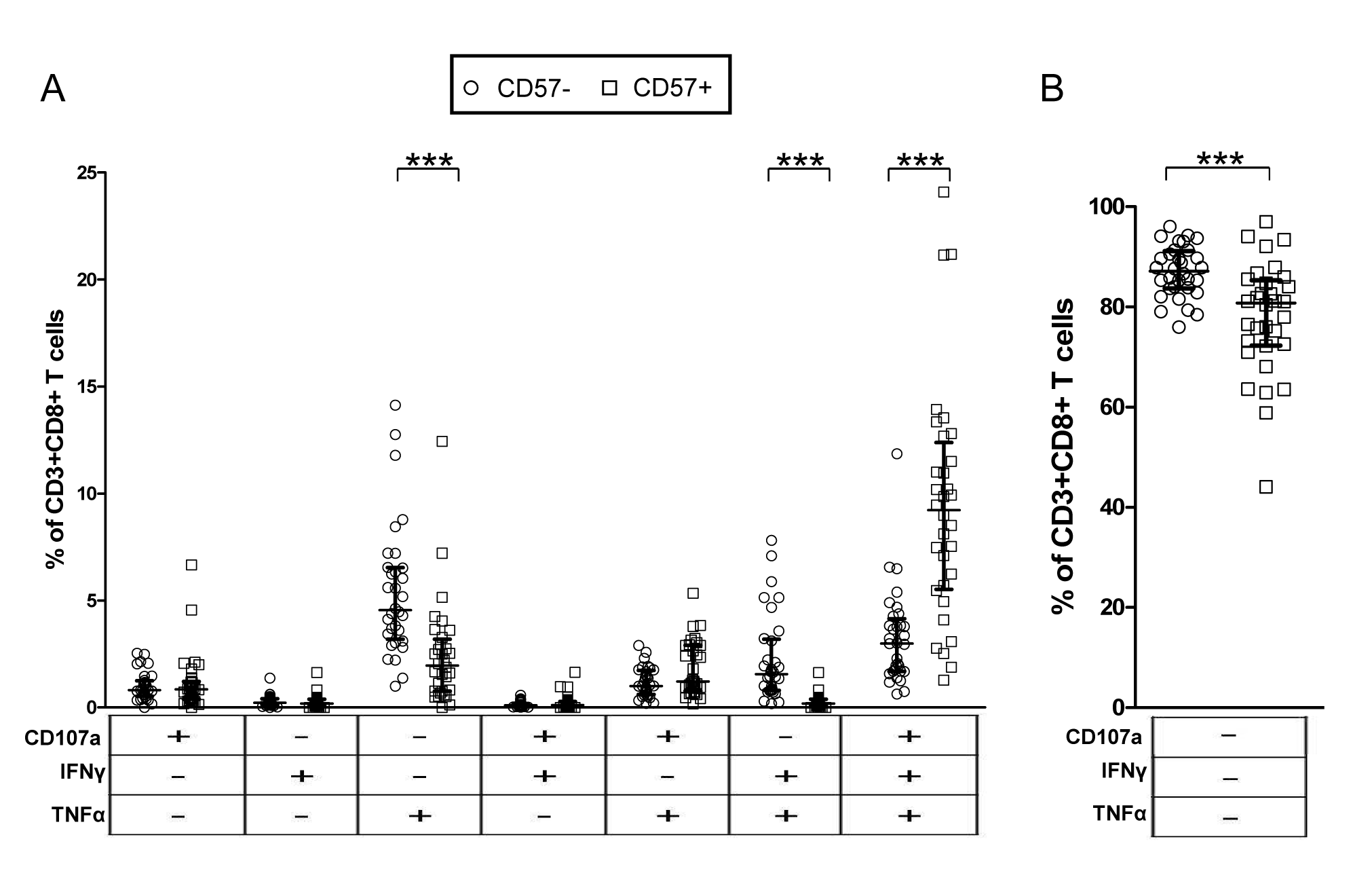

Supplement: Figure S2 — CD8+ T cell polyfunctionality in relation to CD57 expression. Polyfunctionality flow cytometry analysis of CD8+CD57– and CD8+CD57+ T cell subsets, in response to SEB stimulation. This figure summarizes data for all 32 healthy individuals enrolled in this study. Scatter graphs show the magnitude of SEB responses in each functional category, expressed as percentage of CD8+CD57− T cells or CD8+CD57+ T cells. Vertical black lines indicate interquartile ranges, ranging from the 25th to the 75th percentile. The median response for each category is indicated by a horizontal black line. The combination of functions studied is indicated in the table below the scatter graphs. Panel A, cells responding to SEB stimulation. Panel B cells that do not respond to SEB stimulation. (TIF) [file pone.0088538.s002.tif]

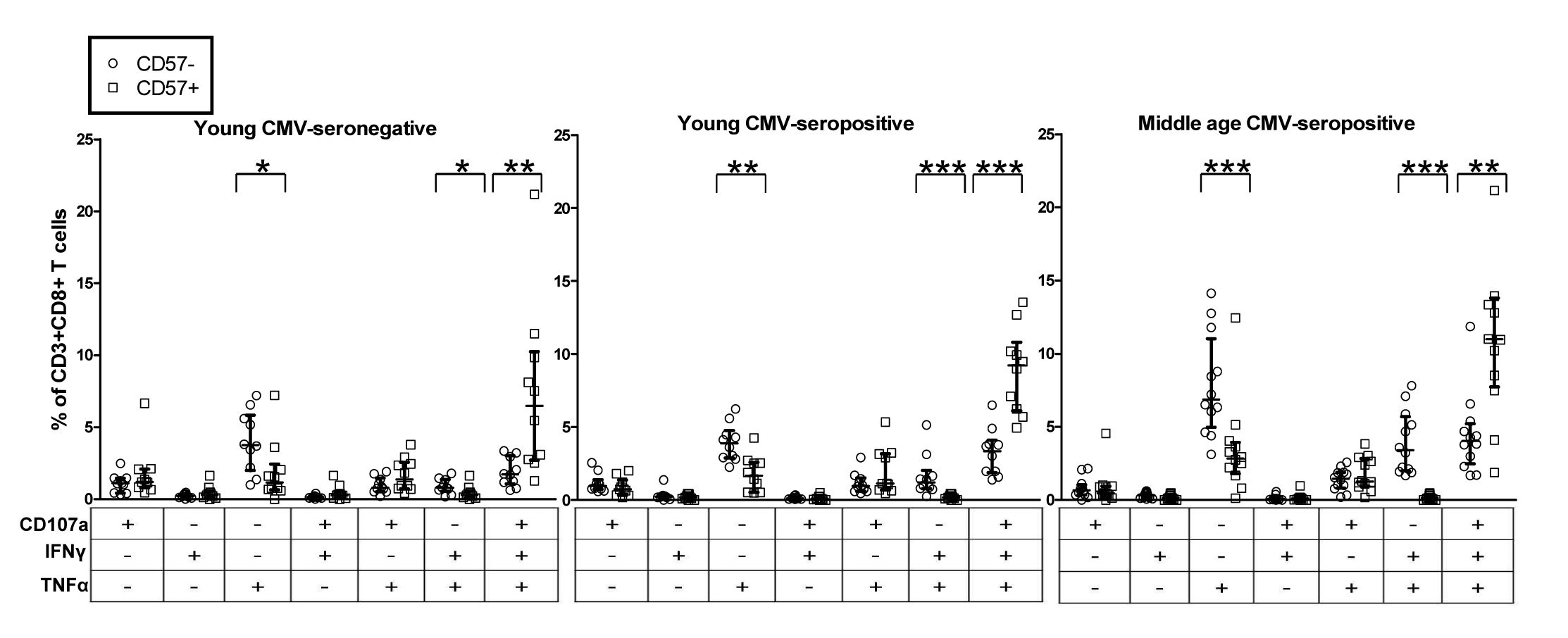

Supplement: Figure S3 — CD8+ T cell polyfunctionality, in relation to CD57 expression, in the different groups. Each graph shows the polyfunctional responses to SEB of CD8+CD57– and CD8+CD57+ T cell subsets for each group studied (young CMV-seronegative, young CMV-seropositive and middle age CMV-seropositive). Scatter graphs show the magnitude of SEB responses in each functional category, expressed as percentage of CD8+CD57− T cells or CD8+CD57+ T cells. Vertical black lines indicate interquartile ranges, ranging from the 25th to the 75th percentile. The median response for each category is indicated by a horizontal black line. The combination of functions studied is indicated in the table below the scatter graphs. (TIF) [file pone.0088538.s003.tif]
